# Effect of Black Rice Starch on Structure and Physical–Mechanical Properties of Carboxymethyl Chitosan/Gellan Gum-Based Intelligent Food Packaging Film and Application in Monitoring Shrimp Freshness

**DOI:** 10.3390/polym18121505

**Published:** 2026-06-16

**Authors:** Siti Ayu Ulfadillah, I-Lin Tsai, Chi Lin, Yu-Hao Huang, Yi-Cheng Ho, Min-Lang Tsai, Fwu-Long Mi

**Affiliations:** 1 Department of Food Science, National Taiwan Ocean University, Keelung 202301, Taiwan; sitiayuulfadillah@gmail.com (S.A.U.); jeffsnsd@gmail.com (Y.-H.H.); 2 Department of Biochemistry and Molecular Cell Biology, School of Medicine, College of Medicine, Taipei Medical University, Taipei City 11031, Taiwan; isabel10@tmu.edu.tw (I.-L.T.); mervynlin11@gmail.com (C.L.); 3 Graduate Institute of Medical Sciences, College of Medicine, Taipei Medical University, Taipei City 11031, Taiwan; 4 Graduate Institute of Nanomedicine and Medical Engineering, College of Biomedical Engineering, Taipei Medical University, New Taipei City 235603, Taiwan; 5 Department of Bioagricultural Science, National Chiayi University, Chiayi 60004, Taiwan; ichengho@mail.ncyu.edu.tw

**Keywords:** intelligent packaging materials, anthocyanins, carboxymethyl chitosan, low-acyl gellan gum, black rice

## Abstract

Visual freshness monitoring is challenging in intelligent seafood packaging. This study developed low-acyl gellan gum (LGG)-based intelligent films incorporating anthocyanin (BRE), carboxymethyl chitosan (CMCh), and black rice starch (BRS) and evaluated their effects on film structure, physical–mechanical properties, and shrimp freshness-monitoring performance. Films prepared via solution casting were evaluated using structural, mechanical, and barrier analyses, alongside shrimp spoilage trials at 25 °C. Structural analyses revealed an integrated polysaccharide network. CMCh reinforced the matrix and increased tensile strength, whereas partially retained BRS granules introduced microstructural heterogeneity, reducing strength and increasing water vapor permeability, highlighting a trade-off between mechanical performance and moisture transport. Consequently, BRS-containing films reduced BRE release, improved pigment retention, and resulted in less intense color changes associated with total volatile basic nitrogen (TVB-N) accumulation during shrimp spoilage. Overall, these results suggest that CMCh and BRS composition-dependently modulate the structure, water vapor transport, pigment retention, and colorimetric response of LGG-based films for visual monitoring of shrimp freshness under accelerated spoilage conditions.

## 1. Introduction

The increasing demand for sustainable, safe, and information-rich food packaging has accelerated the development of biodegradable intelligent packaging systems as alternatives to conventional petroleum-based plastics [1,2]. In this context, polysaccharide-based edible films have attracted considerable attention owing to their renewability, environmental compatibility, and functional versatility [3,4]. As these films are increasingly expected to facilitate visual freshness monitoring, managing their hydration-related behavior under practical storage conditions becomes a key challenge [5,6]. Finding suitable seafood packaging remains challenging due to moisture-rich environments, which may affect film integrity and compromise the stability of colorimetric indicators [5]. Therefore, intelligent films for seafood freshness monitoring require a film matrix that balances mechanical integrity, water vapor transport, pigment retention, and an interpretable color response.

To withstand the stringent conditions of packaging microenvironment and advance environmental sustainability, it is increasingly urgent to develop biopolymer-based matrices that combine mechanical robustness with moisture-related properties for intelligent packaging films. Polysaccharides (e.g., cellulose, starch, chitosan, and gums) are widely explored for their abundance, biocompatibility, and non-toxicity, and they provide versatile platforms for constructing intelligent packaging systems [7]. Beyond structural support, polysaccharide matrices can also host natural functional additives (e.g., pH-responsive pigments), enabling preservation and intelligent freshness indication. Low-acyl gellan gum (LGG) is regarded as a promising film-forming biopolymer because of its high transparency, thermal stability, and ability to form strong gel networks through ordered double-helical aggregation [8,9]. Despite these advantages, films prepared from pristine LGG typically suffer from brittleness and high moisture sensitivity, which significantly limit their practical application in food packaging [10,11]. To overcome these drawbacks, LGG has been blended with other polysaccharides [10,12,13,14], proteins [15,16,17], and inorganic compounds or nanoparticles [12,18,19,20] to enhance its mechanical and functional performance.

Chitosan is a natural cationic polymer with excellent properties. However, the poor water solubility and cationic nature of chitosan limit direct compatibility with LGG, as they tend to form insoluble precipitates. Carboxymethyl chitosan (CMCh) is an amphiprotic chitosan derivative containing amino, carboxyl, and hydroxyl groups. Owing to these functionalities, CMCh shows good molecular compatibility with anionic biopolymers and is well suited for fabricating reinforced composite films with anionic polysaccharides such as LGG [21]. Electrostatic interactions between the carboxylate groups of LGG and the protonated amino groups of CMCh, together with extensive hydrogen bonding, promote the formation of dense polyelectrolyte complexes. These interactions could effectively restrict polymer chain mobility, disrupt crystalline ordering, and significantly enhance film integrity [22].

In parallel, black rice starch (BRS) has gained interest as a natural, low-cost polysaccharide additive that can further modulate film microstructure and barrier-related performance. During thermal processing, starch gelatinization and amylose chain rearrangement may induce V-type crystallinity via inclusion complexation with surrounding polymer chains [23]. This structural transformation can affect film microstructure, moisture transport characteristics, and the distribution and mobility of anthocyanins within the matrix. Consequently, BRS may improve the retention of anthocyanins within the film system. However, incomplete gelatinization of native starch granules can introduce microstructural heterogeneity, thereby affecting mechanical performance and optical properties [24,25]. Thus, in LGG-based systems, BRS serves as a structural modulator that adjusts the film’s compactness and transport characteristics, thereby enhancing stability and regulating colorimetric response in intelligent packaging applications.

Beyond structural and mechanical performance, the incorporation of intelligent sensing functions, particularly colorimetric indicators that respond to spoilage-related environmental changes, represents a key hallmark of next-generation food packaging. Black rice anthocyanin extract (BRE) is a natural, non-toxic pigment that exhibits pronounced pH-dependent color transitions, making it an attractive indicator for freshness monitoring [26]. From a Green Chemistry and Circular Economy perspective, black rice is particularly appealing because multiple fractions can be valorized within a single packaging formulation. Rather than valorizing a single fraction, co-utilizing the starch-rich fraction (BRS) and the anthocyanin-rich extract (BRE) may enable an additive-minimized system with improved compatibility due to their common botanical origin. Anthocyanins undergo reversible structural transformations from red flavylium cations under acidic conditions to purple or blue quinonoidal bases and yellow-green chalcone forms at alkaline pH [27,28]. These transformations are closely associated with food spoilage processes, particularly in protein-rich products such as seafood, where microbial degradation generates volatile basic compounds [29,30,31]. In these moisture-rich packaging environments, effective visual monitoring requires both strong pigment retention to minimize leaching and sufficient chemical stability to prevent degradation and discoloration. Accordingly, strategies to enhance anthocyanin retention and mitigate degradation are needed, as anthocyanins are inherently prone to migration (diffusion), chemical degradation, and discoloration.

To address these challenges, LGG serves as the primary gel matrix, while CMCh and BRS are incorporated to modulate the film’s microstructure and interaction environment. CMCh may enhance intermolecular interactions with anthocyanins through hydrogen bonding and electrostatic interactions, whereas BRS may influence matrix compactness and reduce pigment mobility through structural modification. The combination of these components is expected to create a modified microenvironment that improves the stability and retention of BRE compared with binary systems. Cui et al. (2023) [32] reported that anthocyanins (ACNs), casein (CA)–dextran (DEX) glycated conjugates (UGCA), and carboxymethyl cellulose (CMC), (ACNs-UGCA-CMC) ternary complexes showed significant protection and stability against anthocyanin degradation compared with the binary system.

Despite previous studies on polysaccharide-based intelligent films, the role of CMCh in the film’s structural evolution and its combined effect with BRS on anthocyanin stability and release behavior in LGG-based systems remains insufficiently understood, particularly regarding microstructure–property relationships and freshness-monitoring performance. In this study, a multi-functional LGG-based composite film incorporating CMCh, BRS, and BRE was developed for intelligent food packaging applications. The effects of CMCh and BRS incorporation on the structural, mechanical, barrier, and colorimetric properties of the films, as well as on BRE retention and freshness-indicating performance, were systematically evaluated.

## 2. Materials and Methods

### 2.1. Materials

Low-acyl gellan gum (LGG) (MW 200–300 kDa) was purchased from Chen Fang Co., Ltd., Taipei, Taiwan. N, O-carboxymethyl chitosan (CMCh) (90% deacetylation degree; Mw: ~300 kDa) was purchased from Toronto Research Chemicals, Toronto, Canada. Black rice was purchased from Royal Grains Industrial Co., Ltd., Taitung, Taiwan.

### 2.2. Preparation of Black Rice Extracts

Black rice samples were first ground into fine powders and sieved through a 100-mesh sieve to ensure uniform particle size. Then, 9.26 g of the prepared black rice powder was mixed with 356 mL of 80% ethanol aqueous solution and stirred continuously for 24 h. After extraction, the mixture was filtered and evaporated under dark conditions at 50 °C using a vacuum rotary evaporator. The supernatant was concentrated under reduced pressure to 56 mL. The concentrate was subsequently diluted with distilled water to a final volume of 300 mL, yielding a black rice extract (BRE) solution. The final extract corresponded to approximately 3.09% (*w*/*v*) black rice powder equivalent (9.26 g black rice powder per 300 mL final volume). The BRE solution was stored at 4 °C to minimize pigment degradation until further use.

### 2.3. Analysis of Anthocyanin Types in Black Rice Extract

The anthocyanin profile of BRE was analyzed using ultra-high-performance liquid chromatography coupled with quadrupole tandem mass spectrometry (UHPLC-MS/MS). The sample was subjected to centrifugation at 12,000 RCF for 15 min to remove particles prior to UHPLC-MS/MS analysis. For the analysis, a 2 μL sample injection volume was employed, and it was conducted using the Waters ACQUITY UPLC system connected to the Xevo TQ-XS triple quadrupole mass spectrometer from Waters Corporation (Milford, MA, USA). A Phenomenex KINETEX C18 column (Torrance, CA, USA; 100 mm × 2.1 mm, 2.6 μm, 100 Å) was chosen to separate the analytes, and the column temperature was maintained at 40 °C. The mobile phase consisted of 0.1% formic acid in water (solvent A) and acetonitrile (solvent B). The gradient was as follows: 0–0.5 min, 1% B; 6.5 min, 100% B; 7.5 min, 100% B; 8 min, 1% B; 12 min, 1% B, with a flow rate of 0.2 mL/min.

The ion source of the mass spectrometer was operated in positive electrospray ionization (ESI) mode, with the following settings: capillary voltage at 3.0 kV, source temperature at 150 °C, desolvation temperature at 450 °C, desolvation gas flow rate at 900 L/Hr, and nebulizer gas flow pressure at 7 Bar. Multiple reaction monitoring was employed for compound detection, and the specific parameters are detailed in Table 1. Data analysis was carried out using MassLynx™ Software (Waters Inc., Milford, MA, USA, V4.2).

### 2.4. Determination of Total Phenolic Content of Black Rice Extract

The total phenolic content of the black rice extract was determined using a spectrophotometric method. A 500 µL sample was mixed with 1 mL of 1 N Folin–Ciocalteu reagent and 1 mL of 7.5% sodium carbonate (Na_2_CO_3_) solution. The mixture was thoroughly mixed and incubated in the dark for 3 h to allow the reaction to proceed. After incubation, the mixture was centrifuged at 3000 rpm for 8 min, and the supernatant was collected.

The absorbance of the supernatant was measured at 760 nm using a UV–visible spectrometer. A calibration curve was prepared using gallic acid (GA) at different concentrations to quantify the phenolic content. The total phenolic content was then calculated by interpolating the sample’s absorbance against the standard curve and expressed accordingly [33].

### 2.5. pH-Sensitive Property of BRE

A BRE solution equivalent to 10 mg extract solids per 100 mL was prepared by diluting the stock black rice extract with distilled water. The absorbance of the BRE solutions at various pH levels (pH 2–13) was then measured using a Hitachi UV-Vis spectrophotometer (U-1900, Tokyo, Japan). Spectral data were recorded by scanning the solutions from 450 nm to 700 nm, capturing the visible spectrum associated with anthocyanin color changes.

### 2.6. Preparation of Composite Films

#### Preparation of LGG/CMCh, LGG/BRE, LGG/CMCh/BRE, and LGG/CMCh/BRE/BRS Composite Films

To prepare LGG/CMCh composite films, LGG (0.6 g) was dissolved in 30 mL of deionized water and stirred at 80 °C for 1 h. CMCh (0.2 g) was dissolved in 20 mL of deionized water and heated at 80 °C for 30 min. The two solutions were subsequently combined at a (*v*/*v*) ratio to achieve a final volume of 50 mL and then degassed in an ultrasonic water bath for 1 h. Then, the solutions were cast into Teflon dishes and dried in a hot-air oven at 50 °C for 12 h to form LGG/CMCh composite films. For the LGG/BRE composite films, 30 mL of deionized water used to dissolve LGG was replaced with a BRE solution. LGG (0.6 g) was dissolved in 30 mL of BRE solution, stirred at 80 °C for 1 h, then degassed in an ultrasonic water bath for 1 h, and cast into Teflon dishes at 50 °C for 12 h. For the LGG/CMCh/BRE composite films, deionized water was replaced with black rice anthocyanin extract, and the subsequent steps were unchanged. For the LGG/CMCh/BRE/BRS composite films, LGG, CMCh, and BRE solutions were prepared following the same procedure as described above. After the solution reached a final volume of 50 mL, 0.5 g of BRS powder was added, mixed, and then cast into Teflon dishes. The dried films were removed from the casting plates and kept in a cold, dry location for subsequent usage [29,34]. The selected composite film formulation was based on preliminary assessments of film-forming feasibility, casting stability, and film integrity, rather than on systematic formulation optimization.

### 2.7. Characterization of Films

#### 2.7.1. Scanning Electron Microscopy (SEM)

The surface morphology of the film samples was analyzed using a scanning electron microscope (Hitachi S-4800, Tokyo, Japan). The films were coated with a thin layer of gold to enhance conductivity using a sputter coater (Hitachi, E1010, Tokyo, Japan). Imaging was conducted at an accelerating voltage of 15 kV to capture detailed structural features.

#### 2.7.2. Fourier-Transform Infrared (FTIR) Analysis

The FTIR spectra of the film samples were obtained with a FTS-155 spectrometer (BIO-RAD, Hercules, CA, USA). The samples were cut into small pieces, then combined with KBr (1:99 *w*/*w*), crushed, and dried at 60 °C for 24 h. They were then compressed into 13 mm pellets under 400 kg/cm^2^ for 10 min. Spectra were obtained within the range of 400–4000 cm^−1^ following background correction using a pure KBr pellet, and functional groups were determined from characteristic absorption peaks [35].

#### 2.7.3. X-Ray Diffraction (XRD) Analysis

X-ray diffraction (XRD) was performed using a diffractometer (Bruker AXS, D2 PHASER A26-X1-A2B0B2C, Karlsruhe, Germany). Gellan gum, carboxymethyl chitosan, black rice powder, and composite films were spread on the sample holder and flattened with a glass slide. Measurements were conducted at 40 kV and 20 mA with a 0.66 mm slit and a 0.5 mm mask, scanning from 5° to 40° (2θ) at a rate of 0.5°/s [36].

#### 2.7.4. Assessment of Film Thickness

The thickness of composite films was measured with a micrometer (Peacock G-6C, Tokyo, Japan). To account for potential variations, measurements were taken at 10–20 random points across each film, and the mean value was recorded [37].

#### 2.7.5. Mechanical Properties

The tensile strength (TS) and elongation at break (EB, %) of the film samples were measured using a Texture Analyzer (Horn Instrument Co, Ltd. RapidTA+, New Taipei City, Taiwan). According to Wang et al. (2022) [38], with slight modification, the films were cut into rectangular specimens (approximately 3 cm × 1 cm). The initial grip separation was set to 20 mm, and the crosshead speed was maintained at 2 mm/s. To ensure accuracy, each measurement was performed in triplicate, and the average values were reported.

#### 2.7.6. Moisture Content

Film samples were cut into appropriate sizes and weighed to obtain the wet weight (Wwet). The samples were then dried in an oven at 105 °C for 1 h and reweighed to determine the dry weight (Wdry). The moisture content was calculated using the following equation:

Moisture content (%) = [(Wwet − Wdry)/Wwet] × 100

#### 2.7.7. Water Vapor Permeability (WVP)

The water vapor permeability (WVP) of the film samples was evaluated in accordance with ASTM E96-95. In this test, films were sealed on the top of test cups (8.5 cm in depth and 3.5 cm in diameter) containing 10 mL deionized water and placed in a controlled environment chamber maintained at 25 °C and 40% relative humidity. The weight of each cup was recorded for 24 h to track the weight gain caused by water vapor absorption (Pastor et al., 2013) [39]. The WVP of the films was determined using the following equation:

WVP = Δm × x/(S × ΔP × t)

where Δm (g) represents the weight gain of the cup, x (mm) is the average thickness of the film, S (m^2^) denotes the test area of the film, t (s) is the test time, and ΔP is the vapor pressure differential across the film.

#### 2.7.8. Optical Properties

The transmission spectra of the film samples were measured using a UV–Vis spectrophotometer over the range 200–800 nm. The opacity of the film samples was calculated according to the following equation [40]:

Opacity = A600/χ

where A600 was the absorbance of the film sample at 600 nm, and χ was the film thickness (mm).

### 2.8. pH-Responsive Short-Term Release Test

Composite films were cut into squares (1 cm × 1 cm) and immersed in buffer solutions of varying pH values (pH 2–pH 13). Samples were collected at 10 and 30 min, and the full-wavelength absorbance spectra of the solutions were measured using a multimode microplate reader within the range of 380–800 nm. The release of anthocyanins from the composite films under different pH conditions was then compared.

### 2.9. Time-Dependent Aqueous Release Test

Anthocyanin release was assessed in distilled water at 37 °C with continuous stirring at 60 rpm, following the method described by Hematian et al. (2023) [41], with slight modifications. Briefly, a 1 × 1 cm film specimen was immersed in 2 mL of release medium in a centrifuge tube. At predetermined intervals (0, 0.5, 1, 2, 4, 8, 12, and 24 h), 0.5 mL of the release solution was collected and replaced with an equal volume of fresh medium. Each release sample was separately mixed or diluted with pH 1.0 and pH 4.5 buffers before absorbance measurement at 510 and 700 nm using a UV–Vis spectrophotometer.

The anthocyanin content was calculated according to the following formula (Fuleki & Francis, 1968; Rui et al., 2011) [42,43]:

C = ([(A510 − A700) pH_1.0_ − (A510 − A700) pH_4.5_] × MW × DF × L) ÷ ε

where C is the concentration of anthocyanin (mg/mL), DF is the dilution factor, Mw and ε are the molecular weight and molar absorption coefficient of the anthocyanin, respectively. Because cyanidin-3-glucoside is the dominant anthocyanin in BRE, its molecular weight (Mw) of 449.2 g/mol is used in the present study, and ε is set to 29,600 L/mol × cm.

The release of anthocyanin from the films was estimated using the following equation:

Mt = Ct × V0 + vs. × ΣCi

Cumulative release (%) = (M_t_/M_0_) × 100

where Mt is the cumulative amount of anthocyanin released at time t; Ct is the anthocyanin concentration in the release medium at time t; V0 is the total release volume; vs. is the sampling volume; ΣCi is the sum of anthocyanin concentrations measured at all previous sampling time points; and M0 is the total anthocyanin amount initially loaded in the film

### 2.10. Monitoring Shrimp Freshness with the Indicator Films

Fresh shrimp samples were placed in transparent culture dishes of appropriate size. Composite films were cut to fit and affixed to the inner surface of the dish lids. The dishes were sealed tightly, ensuring that the films did not come into direct contact with the shrimp. The samples were then incubated at 25 °C for 0, 4, 8, 12, and 24 h. During storage, photographs were taken at designated intervals to monitor color changes in the composite films as an indicator of shrimp freshness. Storage at 25 °C served as an accelerated spoilage model to rapidly generate volatile basic compounds and to evaluate the colorimetric responsiveness of the films. This condition was not intended to simulate cold-chain storage.

### 2.11. Determination of Total Volatile Basic Nitrogen (TVB-N)

The total volatile basic nitrogen (TVB-N) content of shrimp samples during storage was determined using the Conway microdiffusion method. Finely minced shrimp muscle (1–2 g) was homogenized with 2.2% (*w*/*v*) trichloroacetic acid (TCA) solution for 10 min. The mixture was filtered, and the filtrate was adjusted to a final volume of 20 mL with TCA solution. For analysis, a Conway microdiffusion dish was prepared by applying a thin layer of Vaseline along the joint between the rim and the lid to ensure airtight sealing. 1 mL of boric acid absorbent solution was placed in the inner chamber, while 1 mL of saturated potassium carbonate solution was pipetted into one side of the outer chamber. Subsequently, 1 mL of the prepared sample extract was added to the opposite side of the outer chamber. The dish was immediately sealed and fixed with a clamp. The contents of the outer chamber were gently mixed by tilting, ensuring that no overflow occurred into the inner chamber. The Conway dish was incubated at 37 °C for 90 min, after which the inner solution was titrated with 0.01 N hydrochloric acid at room temperature, gently stirred with a glass rod. A faint pink coloration of the inner solution indicated the endpoint. A blank test was conducted under identical conditions, replacing the sample solution with distilled water. The TVB-N content was calculated using the following equation:

TVB-N (mg/100 g) = [(C − B) × f × 0.14 × V × 100]/(W × 10)

where C was volume of 0.01 N HCl solution consumed for titration of the sample solution (mL), B was volume of 0.01 N HCl solution consumed for titration of the blank (mL), f was factor (titer) of the 0.01 N HCl solution, 0.14 was equivalent of 1 mL of 0.01 N HCl, corresponding to 0.14 mg of TVB-N, V was final volume of the sample solution after dilution (20 mL), and W was weight of shrimp muscle used for analysis (g)

### 2.12. Color Response to pH

To evaluate the color response of the film samples, they were cut into 20 × 20 mm squares and recorded using a digital camera. The color parameters, including L* (lightness), a* (redness-greenness), and b* (yellowness-blueness), were measured using a colorimeter (Abis WT-5D20, New Taipei City, Taiwan). The total color difference (ΔE) was calculated relative to the initial color of the same film sample before treatment/exposure, using the corresponding L*, a*, and b* values as the reference, according to the following equation:

ΔE = [(L* − L0)2 + (a* − a0)2 + (b* − b0)2]^2^

where L0, a0, and b0 are the color values before the color change; L*, a*, and b* are the color values after the color change.

### 2.13. Statistical Analysis

Data were analyzed by one-way ANOVA, and significant differences among groups were further evaluated using Duncan’s New Multiple Range Test (DMRT). Differences were considered significant at *p* < 0.05. Analyses were performed using IBM SPSS Statistics for Windows, Version 22.0 (IBM Corp., Armonk, NY, USA).

## 3. Results and Discussions

### 3.1. Identification of BRE

**Figure 1:**
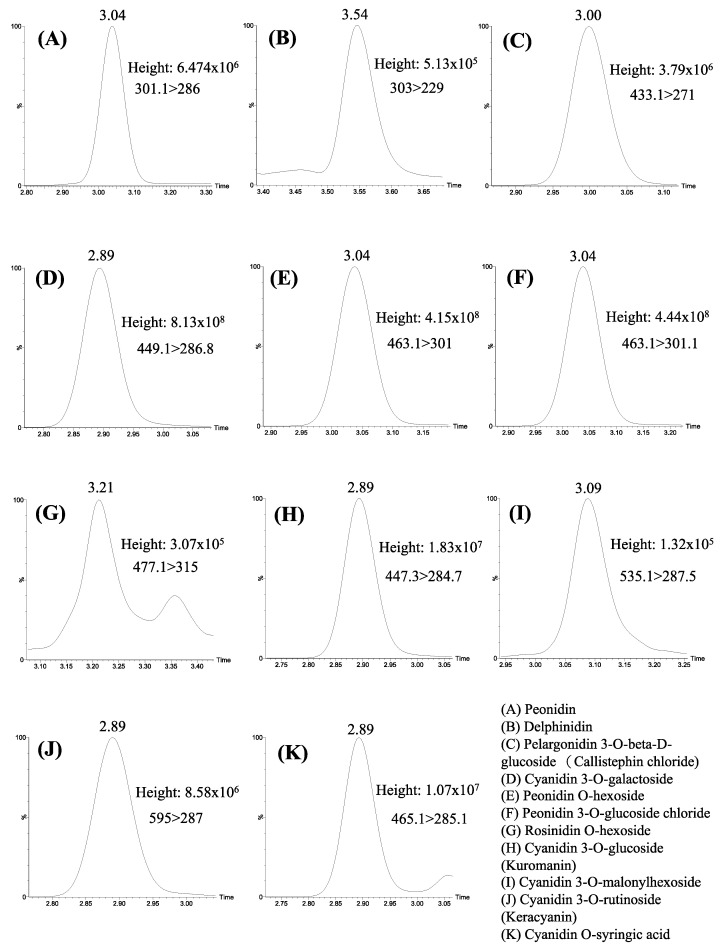
High-performance liquid chromatography–mass spectrometry (HPLC-MS) analysis of the black rice anthocyanin extract (BRE) phytochemical composition.

The identification of black rice anthocyanins in BRE enhances the understanding of black rice’s phytochemical composition. The HPLC-MS analysis confirmed that cyanidin-3-glucoside was the predominant anthocyanin, consistent with previous studies highlighting its abundance in black rice [44,45]. As shown in Figure 1 and Table 1, BRE was mainly composed of anthocyanins, including peonidin, delphinidin, pelargonidin-3-O-glucoside, cyanidin-3-O-galactoside, peonidin O-hexoside, peonidin-3-O-glucoside chloride, osinidin O-hexoside, cyanidin-3-O-glucoside, cyanidin-3-O-malonylhexoside, cyanidin 3-O-rutinoside, and cyanidin O-syringic acid. The presence of these diverse anthocyanins suggests that black rice may offer substantial benefits, including various health-promoting properties [44]. The composition of black rice anthocyanins, particularly the high levels of cyanidin derivatives, has been associated with antioxidative, anti-inflammatory, and potential disease-preventive effects. Furthermore, the chemical diversity observed in BRE indicates its potential applications in functional foods and nutraceutical formulations [46].

### 3.2. Color Responses of BRE Solution

**Figure 2:**
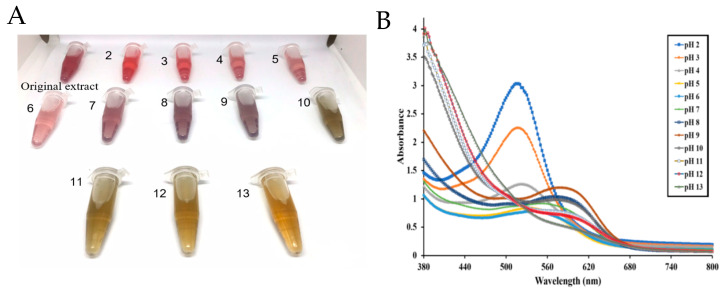
Anthocyanins from black rice extract. (**A**) Color of black rice extract solution containing anthocyanins at different pH levels (pH 2–13). (**B**) UV-vis spectra of black rice anthocyanin solution at different pH levels (pH 2–13).

The pH sensitivity of anthocyanins in BRE and the resulting color changes were clearly demonstrated in our study (Figure 2A). Anthocyanin solutions exhibited distinct color transitions across the pH range of 1.0 to 13.0, including red, pink, violet, blue, green, and yellow. These color variations in BRE across different pH buffer solutions are attributed to the structural transformations of anthocyanins [28]. Under acidic conditions (pH 1.0–3.0), the solutions exhibited a bright red color, attributed to the flavylium cation form of anthocyanins. As the pH increased to the slightly acidic to neutral range (pH 4.0–6.0), the color intensity decreased, appearing pale pink or light purple. This fading is caused by the hydration of the flavylium cation into the colorless carbinol pseudo-base and its equilibrium with the colorless chalcone form. In the pH range of 7.0–9.0, the solutions appeared violet-blue, reflecting the deprotonation of the flavylium cation into the quinonoidal anhydro base. This blue transition is particularly significant, as it serves as a visual indicator of shrimp spoilage, where the accumulation of volatile basic nitrogen (TVB-N) creates an alkaline microenvironment. At pH levels above 10, the color transitioned to brown-green and finally to bright yellow, corresponding to the ionized chalcone form of anthocyanins’ structure degradation. These findings confirm the structural transformations anthocyanins undergo in response to pH changes, offering valuable insights into their stability and potential applications in intelligent food packaging.

Figure 2B illustrates the UV–vis spectra of BRE across varying pH levels, shedding light on the spectral behavior of anthocyanins under different conditions. The analysis revealed distinct absorption peaks at specific pH values, indicative of structural transformations in anthocyanins [47]. At pH 2.0 and 3.0, the maximum absorption peaks were observed around 518 nm. This high absorbance is characteristic of the flavylium cation, the predominant and most stable colored species in acidic environments. As the pH increased to 4.0, a significant hypochromic effect was observed, with the maximum absorption wavelength shifting slightly to 524 nm and the intensity dropping sharply. This trend continued through pH 5.0 and 6.0, where absorbance reached its minimum. This spectral bleaching is attributed to the nucleophilic attack of water on the flavylium cation, forming the colorless carbinol pseudo-base and its equilibrium with the colorless chalcone form. Upon moving to neutral and slightly alkaline conditions (pH 7.0–10.0), the spectra displayed a pronounced bathochromic shift. The absorption shifted from 554 nm at pH 7.0 to 572–580 nm at pH 8.0–10.0. This shift to longer wavelengths is caused by the deprotonation of the anthocyanin’s phenolic hydroxyl groups, resulting in the formation of quinonoidal bases. These structures exhibit extended electron delocalization, which reduces the excitation energy and gives rise to the observed violet and blue colors. At highly alkaline pH (11.0–13.0), the absorbance at 580 nm decreased progressively. Simultaneously, an increase in absorbance was noted at shorter wavelengths (near 400 nm), corresponding to the degradation of the quinonoidal structure into the ionized chalcone. This transition explains the final shift to a yellow hue. These spectral findings align perfectly with the visual color transitions of the BRE solution, confirming its reliability as a colorimetric indicator for pH-responsive intelligent packaging.

### 3.3. Gelation Properties Analysis

The gelation temperatures of LGG, LGG/CMCh, and LGG/CMCh/BRS solutions provide some insight into the intermolecular interactions between the polysaccharides (Supplementary Material, Table S1). The observed gelation transition of LGG hydrogels at 30.64 ± 0.38 °C and a melting temperature of 56.64 ± 4.01 °C aligns with prior findings [13,48]. This gelation transition is attributed to the thermally induced transition of LGG chains from a random coil to an ordered double-helix structure, followed by the aggregation of these helices into a three-dimensional network [49]. This temperature is significantly lower than that of high-acyl GG (46–55 °C) previously reported [49], demonstrating the influence of acyl content on gel stability. The elevated gelation temperature of high-acyl GG is due to the enhanced stability of its double-helix structure, reinforced by the formation of additional hydrogen bonds within and between the participating strands [50]. The incorporation of CMCh into the LGG solution significantly decreased the gelation temperature to 28.14 ± 2.10 °C and the melting temperature to 25.10 ± 1.80 °C. This reduction in thermal stability is likely due to the interference of CMCh chains with the structured conformational transition of LGG. While electrostatic interactions occur, the bulky CMCh chains significantly disrupt the aggregation of LGG double-helices, thereby hindering the formation of the structured junction zones necessary for gelation. Thus, a lower temperature is necessary to overcome this steric hindrance and initiate the transition to an organized gel state. The statement was supported by a study by [51], which found that incorporating CMCh into LGG reduced the gelation temperature. This reduction is attributable to CMCh altering electrostatic repulsion and intermolecular interactions among gellan chains, thereby decreasing the gelation temperature.

In contrast, the addition of BRS significantly elevated the gelation temperatures. For the LGG/BRS system, the temperature increased to 34.65 ± 2.02 °C, while the quaternary LGG/CMCh/BRS system rose to 32.57 ± 0.58 °C. This enhancement may be associated with increased intermolecular hydrogen-bonded network between the starch chains and the LGG/CMCh framework, as suggested by FTIR observations of changes in O–H stretching vibrations [13]. Additionally, the appearance of V-type crystallinity in the composite films suggests that amylose molecules from the starch may form inclusion complexes with the LGG and CMCh chains during processing. These complexes likely act as physical junction points that reinforce the hydrogel’s structural integrity, enabling the network to form at higher temperatures than in starch-free formulations. Interestingly, no measurable melting temperature was observed for the LGG/BRS hydrogel because no distinct gel-to-sol transition occurred within the tested temperature range. While, the LGG/CMCh/BRS system displayed a melting temperature of 34.02 ± 3.84 °C, these results suggest that BRS influences the thermal behavior of the hydrogel network, although the presence of CMCh appears to modify the gel–sol transition characteristics of the composite system. These interactions likely reinforce the hydrogel’s structural integrity, thereby increasing the gelation temperature.

### 3.4. FTIR Analysis

**Figure 3:**
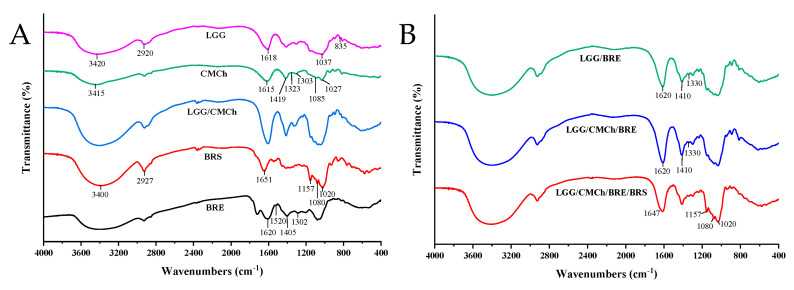
Fourier-transform infrared (FTIR) spectra of (**A**) LGG, CMCh, LGG/CMCh, BRS, and BRE powder. (**B**) LGG/BRE, LGG/CMCh/BRE, and LGG/CMCh/BRE/BRS composite films. Abbreviations: LGG, low-acyl gellan gum; CMCh, carboxymethyl chitosan; BRS, black rice starch; BRE, black rice anthocyanin extract.

FTIR spectroscopy was employed to elucidate the structural features of LGG, CMCh, BRS, and BRE, and to assess intermolecular interactions within the polymer matrix. The spectra of the individual components are presented in Figure 3A, while those of the composite films are shown in Figure 3B. The FTIR spectrum of LGG exhibits characteristic absorption bands at 1618 cm^−1^, attributed to the asymmetric stretching vibration of carboxylate (COO^−^) groups, and at 1037 cm^−1^, corresponding to C–O stretching vibrations of the polysaccharide backbone. A broad band centered at approximately 3420 cm^−1^ is associated with O–H stretching vibrations, reflecting extensive hydrogen bonding, while the absorption at 2920 cm^−1^ arises from aliphatic C–H stretching vibrations [52]. In the case of CMCh, the successful introduction of carboxymethyl groups onto the chitosan backbone is confirmed by several spectral features. A broad absorption band at 3415 cm^−1^ corresponds to overlapping O–H and N–H stretching vibrations. The retention of the amide III band at 1323 cm^−1^ (C–N stretching), together with the emergence of a new band at 1303 cm^−1^ assigned to C–O stretching of carboxymethyl groups, indicates that carboxymethylation occurred without disrupting the primary chitosan structure. This modification is further supported by the appearance of pronounced asymmetric (1615 cm^−1^) and symmetric (1419 cm^−1^) COO^−^ stretching vibrations, which are absent in native chitosan. Additionally, absorption bands in the range of 1085–1027 cm^−1^ are attributed to C–O stretching vibrations of the saccharide rings [10,53].

As shown in Figure 3B, composite films containing BRS exhibit a distinct absorption band at 1647 cm^−1^, which is assigned to the H–O–H bending vibration of bound water associated with the amorphous regions of starch [54,55]. Broad bands in the 3400–3200 cm^−1^ region correspond to O–H stretching vibrations, indicating extensive hydrogen bonding among starch chains and between starch and other polysaccharide components. Characteristic absorption peaks at 1157, 1080, and 1020 cm^−1^ are associated with C–O–C glycosidic bond vibrations and C–OH stretching of secondary alcohol groups, reflecting the fundamental molecular structure of starch [56,57]. Compared with LGG/CMCh film, band O-H shifted from 1651 cm^−1^ to 1647 cm^−1^, which suggests the formation of intermolecular hydrogen bonding between BRS and LGG within the composite matrix [58]. For composite films incorporating BRE (Figure 3B), characteristic anthocyanin-related absorption bands are observed at approximately 1620 cm^−1^ and 1410 cm^−1^, may be attributed to C=C aromatic ring vibrations associated with the flavonoid structure of anthocyanins [28]. Upon incorporation into the polysaccharide matrix, noticeable reductions in peak intensity in the 1330–1650 cm^−1^ region are detected, indicating molecular interactions between anthocyanins and the LGG/CMCh network. These interactions are primarily attributed to hydrogen bonding and secondary interactions between phenolic hydroxyl groups and polysaccharide functional groups [59]. The coexistence of BRS further enhances hydrogen bonding within the system, as evidenced by intensified O–H stretching and glycosidic bond vibrations, which may contribute to improved stabilization of anthocyanins within the composite film matrix. This finding is consistent with previous studies showing that polysaccharides derived from rice starch can form highly stable complexes with black rice anthocyanins, greatly enhancing their thermal and photostability by preventing structural degradation [44]. Similarly, integrating black rice starch (BRS) into our system provides a compatible polysaccharide network that engages in substantial hydrogen-bonding interactions with the BRE, thereby improving the stability and entrapment of the natural colorant in the composite matrix. Finally, large intermolecular interactions cause structural changes of the composite network, thereby limiting the film’s free volume. These spectral changes suggest intermolecular interactions among LGG, CMCh, BRS, and BRE that may influence matrix compactness, pigment retention, and the subsequent colorimetric response.

### 3.5. Thickness and Crystal Property of Composite Films

The analysis of thickness and crystallinity provides valuable insights into the structural properties of the composite films. Table 2 provides a comprehensive analysis of the thickness characteristics of the composite films. The LGG film exhibited the lowest thickness among the tested samples, measuring 33.87 ± 1.76 μm. The addition of BRE and CMCh resulted in notable increase in thickness to 37.12 ± 2.92 μm and 40.55 ± 3.30 μm, respectively. This increase may be due to the additional solid content in the film-forming system, which raises the mass of non-volatile components remaining after drying. Furthermore, interactions between polymer chains and solutes can impact the microstructural organization of the film matrix. In the same study by Yong et al. (2019) [60], adding extract to the film increased its thickness, resulting in a more complex matrix. Significantly, the incorporation of BRS resulted in a marked increase in film thickness to 77.70 ± 2.57 μm, likely attributable to granules within the BRS [61]. This was consistent with the results of SEM observations, which showed that film-contained BRS has a more heterogeneous internal microstructure.

**Figure 4:**
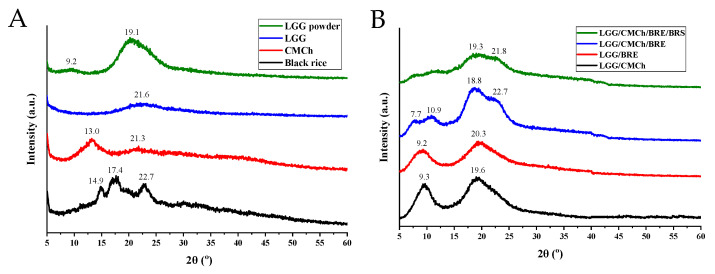
X-ray diffraction (XRD) analysis of (**A**) LGG powder, and LGG, CMCh, and BRS composite films, and (**B**) LGG/CMCh/BRE/BRS, LGG/CMCh/BRE, LGG/BRE, and LGG/CMCh composite films. Abbreviations: LGG, low-acyl gellan gum; CMCh, carboxymethyl chitosan; BRS, black rice starch; BRE, black rice anthocyanin extract.

The crystalline structure and phase transitions of the raw materials and composite films were investigated using XRD (Figure 4). As shown in Figure 4A, the XRD patterns of the raw components are displayed. The raw LGG exhibited characteristic diffraction peaks at 2θ = 9.2° and 19.1°, corresponding to its ordered double-helical structure [58]. However, after heating, melting, and casting into a film, LGG exhibited a broad peak with reduced intensity at approximately 21.6°, indicating a transition to an amorphous and semi-crystalline structure due to the thermal disruption of the helices. CMCh exhibited distinct diffraction peaks at 2θ = 13.0° and 21.3° [62]. However, in the LGG/CMCh composite film, the XRD pattern exhibited a single broad peak between 2θ = 9.3° and 19.6° (Figure 4B). This broadening indicates reduced crystallinity, attributed to molecular interactions between LGG and CMCh. The incorporation of CMCh disrupted LGG’s original hydrogen bonding network by forming new hydrogen bonds between the two polymers. This interference hindered the alignment of LGG chains, thereby reducing crystallinity and altering the structural properties of the composite film.

BRS powder exhibited a clear A-type crystalline diffraction pattern, with characteristic peaks at approximately 2θ = 14.9°, 17.4°, and 22.7° (Figure 4A). Upon integration into the polymer matrix, the characteristic A-type diffraction peaks of BRS became less pronounced and were replaced by a broader halo centered at approximately 2θ = 19.6° (Figure 4B), suggesting disruption of the native starch crystalline order during thermal treatment and film casting. This change may be associated with partial gelatinization and amylose rearrangement within the LGG/CMCh matrix, rather than complete gelatinization of all starch granules. The addition of BRE further affected the film’s microstructure, as evidenced by broadened diffraction patterns and reduced intensities. This suggests that the anthocyanin molecules act as molecular spacers within the matrix. These components form competitive hydrogen bonds with the polysaccharide chains, thereby increasing interchain distance and disrupting long-range crystalline order [28,63]. This observation aligns with previous studies, which indicated that hydrogen bonds formed between polysaccharide components and anthocyanins within GG–anthocyanins and CS–anthocyanins systems [8,9]. Additionally, intermolecular interactions between rice starch and CMCh diminished crystalline regularity in the rice starch-based films [62]. These results highlight a formulation–processing trade-off for BRS: while starch incorporation may retain BRE, microstructural heterogeneity can introduce opacity and reduce color contrast in the intelligent packaging system.

### 3.6. Scanning Electron Microscopy Analysis

**Figure 5:**
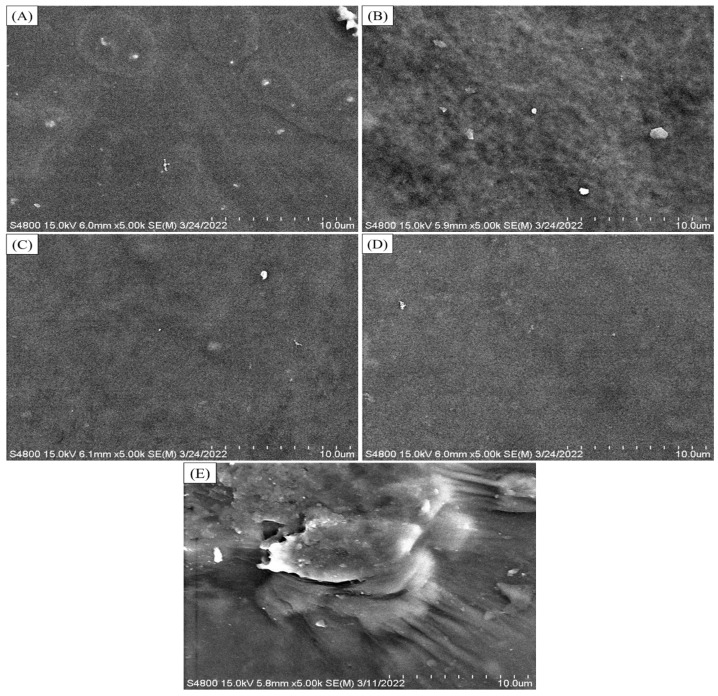
Scanning electron microscopy (SEM) images showing the surface morphology of the composite films at 5000× magnification: (**A**) LGG, (**B**) LGG/CMCh, (**C**) LGG/BRE, (**D**) LGG/CMCh/BRE, and (**E**) LGG/CMCh/BRE/BRS. Abbreviations: LGG, low-acyl gellan gum; CMCh, carboxymethyl chitosan; BRS, black rice starch; BRE, black rice anthocyanin extract.

Figure 5 presents SEM observations indicating that starch-free LGG/CMCh and LGG/CMCh/BRE films exhibited relatively smooth, homogeneous surfaces, suggesting good compatibility among LGG, CMCh, and BRE. In contrast, BRS-containing films displayed partially retained starch granules and a more heterogeneous morphology, indicating incomplete gelatinization or incomplete dispersion of BRS within the LGG/CMCh matrix. This partial gelatinization, despite starch’s inherent hydrophilicity, is attributed to the strong semi-crystalline packing of native granules and to competing hydration by the highly hydrophilic LGG/CMCh matrix, which restricts the availability of free water for complete granular disruption during thermal processing. Starch granules in the endosperm of raw rice are polyhedral and compressed, with proteins distributed along their surfaces [61]. Consistent with previous reports, the observed granules exhibited characteristic polyhedral shapes with distinct angles and typical diameters of 3–8 μm [54]. This heterogeneity was consistent with the reduced tensile strength and increased WVP observed in BRS-containing films. Although these residual granules may contribute to reduced BRE release and delayed color development, their effect should be interpreted as a structural trade-off rather than a purely reinforcing or barrier-enhancing effect.

### 3.7. Mechanical Properties of Composite Films

**Figure 6:**
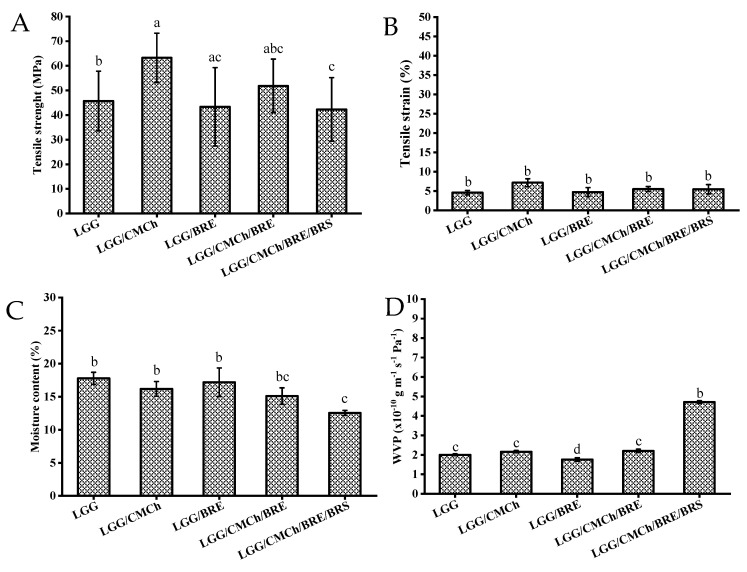
The mechanical and barrier properties of the composite films: (**A**) tensile strength, (**B**) tensile strain, (**C**) moisture content, and (**D**) water vapor permeability (WVP). All data are expressed as mean values ± standard deviation (*n* = 10). Different lowercase letters (a–d) above the bars indicate significant differences between the means obtained by Duncan’s test (*p* < 0.05). Abbreviations: LGG, low-acyl gellan gum; CMCh, carboxymethyl chitosan; BRS, black rice starch; BRE, black rice anthocyanin extract.

The mechanical performance of food packaging films, specifically tensile strength (TS) and elongation at break (EAB), is critical for maintaining structural integrity during food handling and storage. The mechanical parameters for the various film formulations are summarized in Figure 6. As shown in Figure 6A, the incorporation of CMCh into LGG notably enhanced the tensile strength from 45.69 ± 12.12 MPa to 63.24 ± 9.99 MPa, likely due to improved intermolecular interactions, specifically the formation of a polyelectrolyte complex through electrostatic attraction between the anionic groups of LGG and the amino groups of CMCh [64]. This results in a denser, more robust molecular network, as evidenced by the smooth surface morphology observed in the SEM images. However, the addition of BRE and BRS resulted in decreases in TS of 51.81 ± 10.88 MPa and 42.28 ± 12.92 MPa, respectively. The reduced tensile strength of the LGG/CMCh/BRE film compared to the LGG/CMCh film may be attributed to anthocyanin molecules, which may interfere with the hydrogen bonding and electrostatic coupling between the primary polysaccharides. Alizadeh-sani et al. (2021) [65] also found that the addition of anthocyanin decreased the film-based chitosan’s strength and stiffness significantly due to the increased interfacial force between anthocyanin and the polymer matrix through H-bonding formation. For the BRS-containing films, the additional reduction in TS is likely attributable to the presence of semi-gelatinized starch granules. As shown by SEM analysis, these coarse, polyhedral aggregates create discontinuities and act as stress concentrators within the matrix, thereby facilitating premature fracture under mechanical loading [63]. Still, this reduction in macroscopic mechanical strength is a necessary functional trade-off. These rigid, solid microstructures act as a physical barrier; they help regulate and delay the color response of BRS-containing films, which may be associated with reduced BRE release, increased opacity, and altered matrix morphology.

Furthermore, although tensile strength decreased, the elongation at break (EAB) remained largely stable, with no significant differences across all sample alterations (Figure 6B). This indicates that although the addition of BRE and BRS particles introduced stress concentrators that reduced the mechanical resistance, they did not undermine the intrinsic flexibility and plastic deformation capability of the basic LGG/CMCh network.

The moisture content analysis of the composite films highlights the significant influence of composition on water retention properties. The incorporation of CMCh did not significantly reduce the moisture content of the LGG film (Figure 6C). The further addition of BRE/BRS led to a notable decrease in moisture content, which can be attributed to the strong molecular interactions between BRE/BRS and the LGG/CMCh matrix. Furthermore, the interactions between starch in BRS and anthocyanins in BRE suggest that anthocyanins may modify the hydration behavior of the films. These interactions, likely mediated by hydrogen bonding and van der Waals forces, reduce the availability of free hydroxyl groups, thereby lowering overall moisture content [45,57,66]. Overall, these results provide valuable insights into the tunability of moisture content in composite films, allowing for tailored applications in food packaging and biomedical fields where water resistance or retention is a critical factor.

The water vapor permeability (WVP) of the composite films changed notably upon the incorporation of various components. The LGG/CMCh film exhibited a WVP of 2.16 ± 0.06 × 10^−10^ g·m^−1^·s^−1^·Pa^−1^, which slightly increased to 2.20 ± 0.09 × 10^−10^ g·m^−1^·s^−1^·Pa^−1^, indicating a marginal reduction in water vapor barrier performance after BRE incorporation (Figure 6D). This slight increase may be related to changes in matrix continuity and/or hydrophilic domains introduced by the extract components, which could facilitate water vapor diffusion. The subsequent addition of BRS significantly increased the WVP to 4.71 ± 0.07 × 10^−10^ g·m^−1^·s^−1^·Pa^−1^. This marked increase is primarily attributed to the inherent chemical nature of the starch. The partially gelatinized BRS granules introduce numerous hydrophilic hydroxyl groups, thereby increasing the matrix’s overall hydrophilicity and reducing its moisture resistance. Overall, this highlights a composition-dependent trade-off: while BRS introduces microstructural heterogeneity that modulates pigment retention and the resulting colorimetric response, its hydrophilic nature simultaneously reduces the film’s moisture resistance.

### 3.8. Anthocyanin Release Test

**Figure 7:**
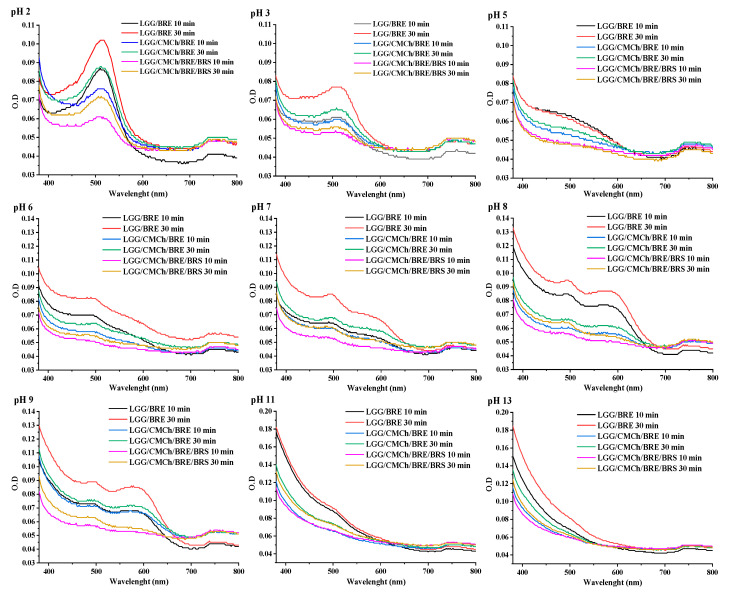
UV-vis spectra of anthocyanin release from the composite films at different pH levels. Abbreviations: LGG, low-acyl gellan gum; CMCh, carboxymethyl chitosan; BRS, black rice starch; BRE, black rice anthocyanin extract.

Figure 7 illustrates the anthocyanin release behavior of different composite films across varying pH conditions at 30 min. The dissolution profile reveals notable differences among the composite films. The LGG/BRE film exhibited the highest anthocyanin release rate at 30 min under all tested pH conditions, suggesting that its structural properties promote rapid anthocyanin diffusion. The stability of the anthocyanins may be a result of the molecular interactions, including hydrogen bonding interactions, between the gellan gum and anthocyanins [28,67]. This behavior may result from the hydrophilic nature of LGG, which enhances anthocyanin solubilization. The LGG/CMCh/BRE film displayed a slower release rate compared to the LGG/BRE film, indicating that the inclusion of CMCh imparted structural integrity and modulated release by influencing film porosity and swelling behavior. CMCh likely contributed to a more gradual dissolution process through hydrogen bonding or electrostatic interactions with LGG [47,60], particularly under different pH conditions.

In contrast, the LGG/CMCh/BRS/BRE film exhibited much lower anthocyanin release at all pH levels, especially at 30 min. The hydroxyl groups in BRS readily formed intermolecular hydrogen bonds with anthocyanins [23]. Instead of increasing permeability, these strong interactions immobilized the anthocyanins within the polymer network. This physical anchoring, combined with a potentially denser film structure, hindered the diffusion of anthocyanins from the film matrix, consequently reducing the release rate of BRE. The time-dependent release profiles illustrated in Figure S1 further support this finding, indicating that BRS-containing films exhibited reduced cumulative anthocyanin release over the entire release period compared with the starch-free formulations. From a practical perspective, the release behavior directly reflects the risk of indicator leaching; therefore, reduced BRE release from BRS-containing films indicates stronger pigment retention within the composite matrix and supports their potential use as colorimetric freshness indicators.

### 3.9. Color Responses of Indicator Films to pH Changes

**Figure 8:**
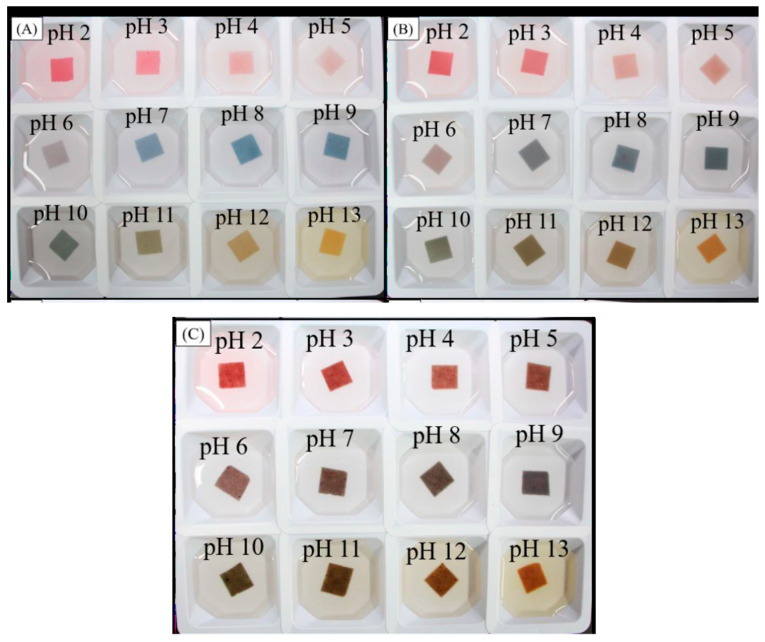
Visual color changes of the (**A**) LGG/BRE, (**B**) LGG/CMCh/BRE, and (**C**) LGG/CMCh/BRE/BRS composite films immersed in different pH buffer solutions. Abbreviations: LGG, low-acyl gellan gum; CMCh, carboxymethyl chitosan; BRS, black rice starch; BRE, black rice anthocyanin extract.

Figure 8 illustrates the visual color changes in films composed of polysaccharides and anthocyanins under various pH conditions, demonstrating the inherent pH-responsiveness of anthocyanin-based systems. Furthermore, quantitative colorimetric analysis (Tables S2–S5) corroborated these visual color shifts. Across all pH levels, the films exhibited a color trend consistent with the anthocyanin extract, indicating the preservation of its natural pH-sensitive properties. However, the film composition significantly impacted the vibrancy and tone of these colors. The LGG/BRE and LGG/CMCh/BRE films showed the highest color vibrancy. The high transparency of the gellan gum and chitosan matrix provides an excellent background for pigment visibility, making the color transitions easy to discern with the naked eye. However, the addition of BRS resulted in a significant decrease in L* (lightness) and a reduction in color contrast, which is consistent with the optical properties of the films (Table S6). This optical trade-off is likely due to the inherent pigments and higher opacity of black rice starch, which mask the subtle color shifts of the added BRE, particularly at neutral pH.

Despite the film’s color becoming darker, the films reliably demonstrated the characteristic structural transformations of anthocyanins across the pH spectrum. At pH 2, high positive a* values confirmed the dominance of the flavylium cation (red). As pH increased, anthocyanins typically fade to pale purple/near colorless around pH 5–7. Greenish tones (negative a*) generally occur under alkaline conditions (~pH 10–13). This transition represents the degradation of anthocyanin structures into chalcones. The substantial changes in L*, a*, and b* parameters confirm the films’ potential as colorimetric indicators, showing clear shifts in chromaticity corresponding to pH variations. The pH-dependent color changes observed in both visual and instrumental analyses highlight the potential of these composite films as practical pH-sensitive materials. Their capacity to reflect pH changes through distinct, measurable color changes underscores their suitability for applications such as intelligent food packaging, where real-time monitoring of product freshness or environmental conditions is essential [68,69]. Moreover, the tunability of color characteristics through the integration of different film components (e.g., BRS) provides a versatile strategy for designing films tailored to specific needs, thereby enhancing their applicability across diverse functional settings.

### 3.10. TVB-N Determination and Analysis

**Figure 9:**
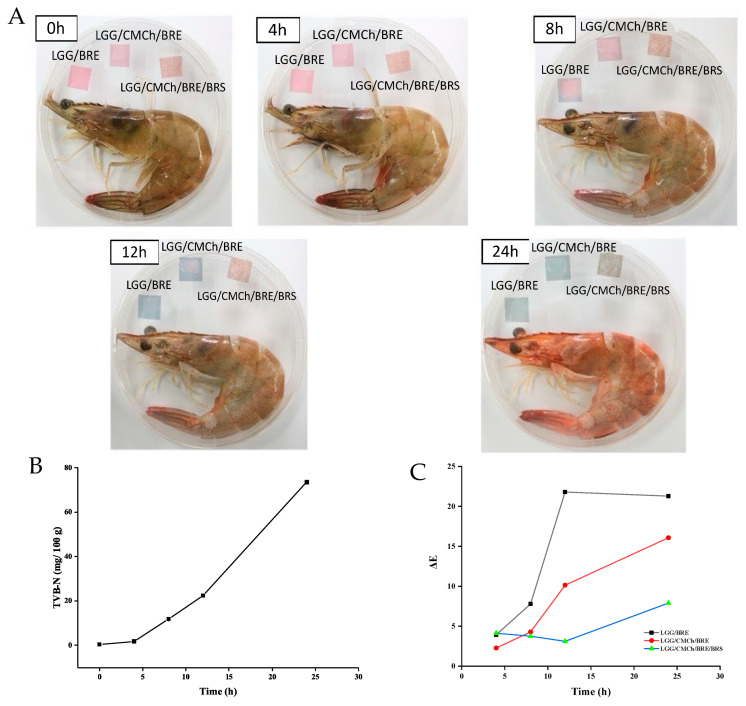
Analysis of composite films in response to shrimp spoilage: (**A**) visual color changes of the composite films present in shrimp samples under storage conditions (25 °C), (**B**) total volatile basic nitrogen (TVB-N) analysis, and (**C**) total color difference. Abbreviations: LGG, low-acyl gellan gum; CMCh, carboxymethyl chitosan; BRS, black rice starch; BRE, black rice anthocyanin extract.

The practical application of the composite films as intelligent indicators was evaluated by monitoring the freshness of white shrimp stored at 25 °C. The effectiveness of the system was assessed by correlating Total Volatile Basic Nitrogen (TVB-N) levels with visual color changes and total color difference (ΔE), as shown in Figure 9. The visual color transitions of the films (Figure 9A) provided a clear indication of the spoilage process and the efficacy of the intelligent packaging system under accelerated storage conditions. As shown in Figure 9B, the initial TVB-N level was 0.34 ± 0.00 mg/100 g, indicating shrimp freshness. During the first 4 h, TVB-N increased slightly to 1.76 ± 0.00 mg/100 g, which remains well within the fresh range. A significant increase was noted at 8 h (11.81 ± 0.20 mg/100 g), but the critical inflection point occurred at 12 h, where TVB-N reached 22.43 ± 0.21 mg/100 g. This level approaches the established spoilage threshold for seafood (25 mg/100 g), signaling the transition from “fresh” to “sub-fresh” or “spoiled.” By 24 h, TVB-N reached a maximum of 73.62 ± 0.91 mg/100 g, indicating advanced microbial decomposition and the accumulation of volatile nitrogenous bases such as ammonia and trimethylamine.

The microstructural changes, as evidenced by FTIR and XRD, provide possible explanations for the composition-dependent colorimetric response, but the response should also be considered together with BRE release, film opacity, and matrix morphology. At the 12 h stage, the LGG/BRE and LGG/CMCh/BRE films exhibited rapid sensitivity, producing pronounced color transitions from pink to blue and a substantial ΔE above 20 (Table S5). During shrimp storage at 25 °C, TVB-N values increased progressively, confirming the development of volatile basic compounds under accelerated spoilage conditions. The BRE-containing films exhibited visible color changes that corresponded to the accumulation of TVB-N. Compared with starch-free films, BRS-containing films showed delayed and less intense color development, which may be associated with reduced BRE release, increased opacity, and heterogeneous matrix morphology (Table S6). Because volatile amine diffusion was not directly measured, this delayed response should be interpreted as formulation-dependent modulation of the colorimetric signal rather than direct evidence of controlled TVB-N permeation.

## 4. Conclusions

In this study, LGG-based intelligent films incorporating CMCh, BRS, and BRE were successfully developed and evaluated. The incorporation of CMCh enhanced the structural integrity of the LGG matrix and increased the tensile strength from 45.69 ± 12.12 to 63.24 ± 9.99 MPa, whereas the addition of BRS increased film thickness from 40.55 ± 3.30 μm to 77.70 ± 2.57 μm and modified the internal microstructure through granule-associated heterogeneity. These structural changes resulted in a composition-dependent trade-off between mechanical performance and moisture barrier properties, with WVP increasing from 2.20 ± 0.09 × 10^−10^ g·m^−1^·s^−1^·Pa^−1^ to 4.71 ± 0.07 × 10^−10^ g·m^−1^·s^−1^·Pa^−1^. Furthermore, incorporating CMCh and BRS reduced BRE release from 47.17% to 32.61%, indicating improved anthocyanin retention within the composite matrix. The BRE-containing films exhibited pH-responsive color transitions and distinct visual color changes during shrimp storage at 25 °C, which corresponded to TVB-N accumulation. Overall, the results demonstrate that CMCh and BRS effectively modulate the structure, moisture-related properties, pigment retention, and freshness-indicating performance of LGG-based intelligent films, highlighting their potential for visual monitoring of shrimp freshness.

## Figures and Tables

**Table 1:** Parameters of multiple reaction monitoring for 11 analytes of BRE.

No.	Name	RT	Cone (V)	Collision Energy (eV)	Parent Ion (*m*/*z*)	Daughter Ion (*m*/*z*)
1	Peonidin	3.04	35	20	301.1	286.0
2	Delphinidin	3.54	35	20	303.0	229.0
3	Pelargonidin 3-O-glucoside (Callistephin chloride)	3.00	35	20	433.1	271.0
4	Cyanidin 3-O-galactoside	2.89	35	20	449.1	286.0
5	Peonidin O-hexoside	3.04	35	20	463.1	301.0
6	Peonidin 3-O-glucoside chloride	3.04	35	20	463.1	301.1
7	Rosinidin O-hexoside	3.21	35	20	477.1	315.0
8	Cyanidin 3-O-glucoside (Kuromanin)	2.89	35	20	493.2	331.0
9	Cyanidin 3-O-malonylhexoside	3.09	35	20	535.1	287.5
10	Cyanidin 3-O-rutinoside (Keracyanin)	2.89	35	20	595.0	287.0
11	Cyanidin O-syringic acid	2.89	35	20	625.4	301.0

Abbreviations: BRE, black rice anthocyanin extract.

**Table 2:** The thickness of LGG, LGG/CMCh, LGG/BRE, LGG/CMCh/BRE, LGG/CMCh/BRE/BRS.

Films	Thickness (μm)
LGG	33.87 ± 1.76 ^d^
LGG/CMCh	40.55 ± 3.30 ^c^
LGG/BRE	37.12 ± 2.92 ^cd^
LGG/CMCh/BRE	43.12 ± 4.39 ^c^
LGG/CMCh/BRE/BRS	77.70 ± 2.57 ^b^

All data are expressed as mean values ± standard deviation (*n* = 10). a–d Different letters in the same column indicate significant differences between the means obtained by Duncan’s test (*p* < 0.05). Abbreviations: LGG, low-acyl gellan gum; CMCh, carboxymethyl chitosan; BRS, black rice starch; BRE, black rice anthocyanin extract.

## Data Availability

The original contributions presented in the study are included in the article; further inquiries can be directed to the corresponding authors.

## Supplementary Materials

The following supporting information can be downloaded at: https://www.mdpi.com/article/10.3390/polym18121505/s1, Table S1: Gelation and gel melting temperature of the LGG, LGG/CMCh, LGG/BRS, and LGG/CMCh/BRS; Table S2: Color parameters (L*, a*, b*) of the LGG/BRE composite film in different pH buffer solutions; Table S3: Color parameters (L*, a*, b*) of the LGG/CMCh/BRE composite film in different pH buffer solutions; Table S4: Color parameters (L*, a*, b*) of the LGG/CMCh/BRE/BRS composite film in different pH buffer solutions; Table S5: Monitoring changes in color parameters (L*, a*, b*) of the composite films in response to shrimp spoilage during the storage time; Table S6: Opacity of composite films. Figure S1: Time-dependent release rate of anthocyanins from LGG-based films in water at different times.

## Abbreviations

The following abbreviations are used in this manuscript:

LGG	Low-Acyl Gellan Gum
LGG/CMCh	Low-Acyl Gellan Gum/Carboxymethyl Chitosan
LGG/BRE	Low-Acyl Gellan Gum/Black Rice Anthocyanins
LGG/CMCh/BRE	Low-Acyl Gellan Gum/Carboxymethyl Chitosan/Black Rice Anthocyanins
LGG/CMCh/BRE/BRS	Low-Acyl Gellan Gum/Carboxymethyl Chitosan/Black Rice Anthocyanins/Black Rice Starch
