# Supplementary material for: Effect of Black Rice Starch on Structure and Physical–Mechanical Properties of Carboxymethyl Chitosan/Gellan Gum-Based Intelligent Food Packaging Film and Application in Monitoring Shrimp Freshness
Source: Polymers (Basel). 2026 Jun 16;18(12):1505. doi: 10.3390/polym18121505 (PMC13306674; doi:10.3390/polym18121505)
Supplement: Supplementary file 1 [file polymers-18-01505-s001.zip › polymers-4341031-supplementary.pdf]

Table S1. Gelation and gel melting temperature of the LGG, LGG/CMCh, LGG/BRS, and LGG/CMCh/BRS.

Hydrogels	Gelation temperature (°C)	Gel melting temperature (°C)
LGG	30.64 ± 0.38[ab]	56.64 ± 4.01[a]
LGG/CMCh	28.14 ± 2.10[b]	25.10 ± 1.80[c]
LGG/BRS	34.65 ± 2.02[a]	-
LGG/CMCh/BRS	32.57 ± 0.58[a]	34.02 ± 3.84[b]

All data were expressed as mean values ± standard deviation (n=3)

[a-c] Different letters in the same column indicate significant differences between the means obtained by Duncan's test ($p<0.05$).

Abbreviations: LGG, low-acyl gellan gum; CMCh, carboxymethyl chitosan; BRS, black rice starch.

Table S2. Color parameters (L*, a*, b*) of the LGG/BRE composite film in different pH buffer solutions.

pH value	L*	a*	b*
2	59.20 ± 0.34	26.94 ± 0.49	28.68 ± 0.27
3	70.10 ± 0.14	16.64 ± 0.14	28.42 ± 0.14
4	77.07 ± 0.13	4.54 ± 0.11	29.23 ± 0.08
5	78.66 ± 0.06	-0.66 ± 0.05	28.15 ± 0.13
6	65.42 ± 0.03	-7.14 ± 0.09	18.56 ± 0.05
7	61.96 ± 0.05	-15.29 ± 0.11	7.91 ± 0.08
8	59.07 ± 0.09	-17.05 ± 0.07	6.37 ± 0.17
9	56.30 ± 0.08	-15.25 ± 0.09	6.79 ± 0.12
10	47.08 ± 0.13	-11.38 ± 0.31	17.55 ± 0.05
11	56.29 ± 0.09	-9.64 ± 0.04	29.92 ± 0.05
12	62.94 ± 0.05	-4.01 ± 0.05	40.06 ± 0.15
13	65.26 ± 0.08	1.21 ± 0.12	54.54 ± 0.06

All data were expressed as mean values ± standard deviation (n=9).
Abbreviations: LGG, low-acyl gellan gum; BRE, black rice anthocyanin extract.

Table S3. Color parameters (L*, a*, b*) of the LGG/CMCh/BRE composite film in different pH buffer solutions.

pH value	L*	a*	b*
2	60.39 ± 0.22	26.83 ± 0.29	31.16 ± 0.14
3	67.34 ± 0.03	16.62 ± 0.14	29.46 ± 0.08
4	75.28 ± 0.07	4.17 ± 0.08	29.58 ± 0.06
5	69.06 ± 0.13	7.63 ± 0.02	30.98 ± 0.18
6	65.74 ± 0.08	-0.77 ± 0.11	23.61 ± 0.13
7	57.96 ± 0.07	-9.24 ± 0.02	16.36 ± 0.12
8	52.56 ± 0.42	-9.44 ± 0.19	12.63 ± 0.025
9	47.17 ± 0.06	-9.06 ± 0.03	10.80 ± 0.07
10	55.56 ± 0.25	-8.51 ± 0.05	25.04 ± 0.08
11	51.31 ± 0.13	-5.40 ± 0.10	30.35 ± 0.07
12	52.84 ± 0.07	-0.39 ± 0.14	35.27 ± 0.09
13	58.59 ± 0.09	8.99 ± 0.09	46.34 ± 0.03

All data were expressed as mean values ± standard deviation (n=9).
Abbreviations: LGG, low-acyl gellan gum; CMCh, carboxymethyl chitosan; BRE, black rice anthocyanin extract.

Table S4. Color parameters (L*, a*, b*) of the LGG/CMCh/BRE/BRS composite film in different pH buffer solutions.

pH value	L*	a*	b*
2	48.84 ± 0.28	35.35 ± 0.11	34.45 ± 0.10
3	47.91 ± 0.21	27.57 ± 0.16	30.78 ± 0.09
4	52.96 ± 0.25	18.60 ± 0.04	30.49 ± 0.07
5	45.54 ± 0.19	16.01 ± 0.15	27.33 ± 0.04
6	53.83 ± 0.11	4.39 ± 0.07	25.02 ± 0.10
7	44.75 ± 0.06	1.66 ± 0.04	23.26 ± 0.04
8	43.05 ± 0.28	-1.26 ± 0.07	21.39 ± 0.08
9	42.57 ± 0.34	-2.63 ± 0.12	15.35 ± 0.33
10	39.62 ± 0.11	-1.62 ± 0.11	27.68 ± 0.05
11	37.93 ± 0.43	2.65 ± 0.15	27.71 ± 0.30
12	42.40 ± 0.49	9.07 ± 0.11	33.29 ± 0.24
13	45.13 ± 0.15	17.62 ± 0.08	38.08 ± 0.13

All data were expressed as mean values ± standard deviation (n=9).
Abbreviations: LGG, low-acyl gellan gum; CMCh, carboxymethyl chitosan; BRS, black rice starch; BRE, black rice anthocyanin extract.

Table S5. Monitoring changes in color parameters (L*, a*, b*) of the composite films in response to shrimp spoilage during the storage time.

Samples	Time (h)	L*	a*	b*	ΔE
LGG/BRE	0	83.03 ± 0.24	2.80 ± 0.16	24.54 ± 0.05	-
	4	86.36 ± 0.16	1.55 ± 0.03	26.19 ± 0.05	3.92 ± 0.15
	8	85.70 ± 0.41	-4.37 ± 1.06	26.02 ± 1.70	7.80 ± 0.93
	12	81.10 ± 0.09	-17.51 ± 0.06	16.85 ± 0.08	21.80 ± 0.18
	24	85.13 ± 0.64	-18.25 ± 0.14	22.21 ± 0.26	21.28 ± 0.45
LGG/CMCh/BRE	0	85.94 ± 0.12	-3.24 ± 0.13	23.76 ± 0.06	-
	4	87.79 ± 0.08	-2.78 ± 0.17	25.00 ± 0.17	2.27 ± 0.12
	8	82.43 ± 0.16	-3.35 ± 0.02	26.21 ± 0.09	4.28 ± 0.11
	12	84.32 ± 0.18	-12.51 ± 0.34	19.97 ± 0.32	10.14 ± 0.34
	24	83.27 ± 0.09	-18.55 ± 0.05	19.64 ± 0.18	16.08 ± 0.15
LGG/CMCh/BRE/BRS	0	80.54 ± 0.21	-3.64 ± 0.17	30.06 ± 0.13	-
	4	84.02 ± 0.23	-3.09 ± 0.11	32.21 ± 0.26	4.13 ± 0.14
	8	78.06 ± 0.25	-3.06 ± 0.17	32.80 ± 0.11	3.74 ± 0.04
	12	83.46 ± 0.58	-4.05 ± 0.34	31.03 ± 0.09	3.10 ± 0.41
	24	78.63 ± 0.59	-10.97 ± 0.22	27.85 ± 0.35	7.89 ± 0.44

All data were expressed as mean values ± standard deviation (n=9).
Abbreviations: LGG, low-acyl gellan gum; CMCh, carboxymethyl chitosan; BRS, black rice starch; BRE, black rice anthocyanin extract.

Table S6. Opacity of composite films.

Composite films	Opacity (mm^{-1})	Transmittance (%)
LGG	3.26 ± 0.002^{a}	30.67 ± 0.002^{a}
LGG/CMCh	3.21 ± 0.003^{a}	31.15 ± 0.003^{a}
LGG/BRE	18.36 ± 0.58^{ab}	5.45 ± 0.58^{ab}
LGG/CMCh/BRE	26.31 ± 0.35^{b}	3.80 ± 0.35^{b}
LGG/CMCh/BRE/BRS	38.67 ± 0.03^{c}	2.59 ± 0.03^{c}

All data were expressed as mean values ± standard deviation (n=3)

$^{a-c}$ Different letters in the same column indicate significant differences between the means obtained by Duncan's test ($p<0.05$).

Abbreviations: LGG, low-acyl gellan gum; CMCh, carboxymethyl chitosan; BRS, black rice starch; BRE, black rice anthocyanin extract.

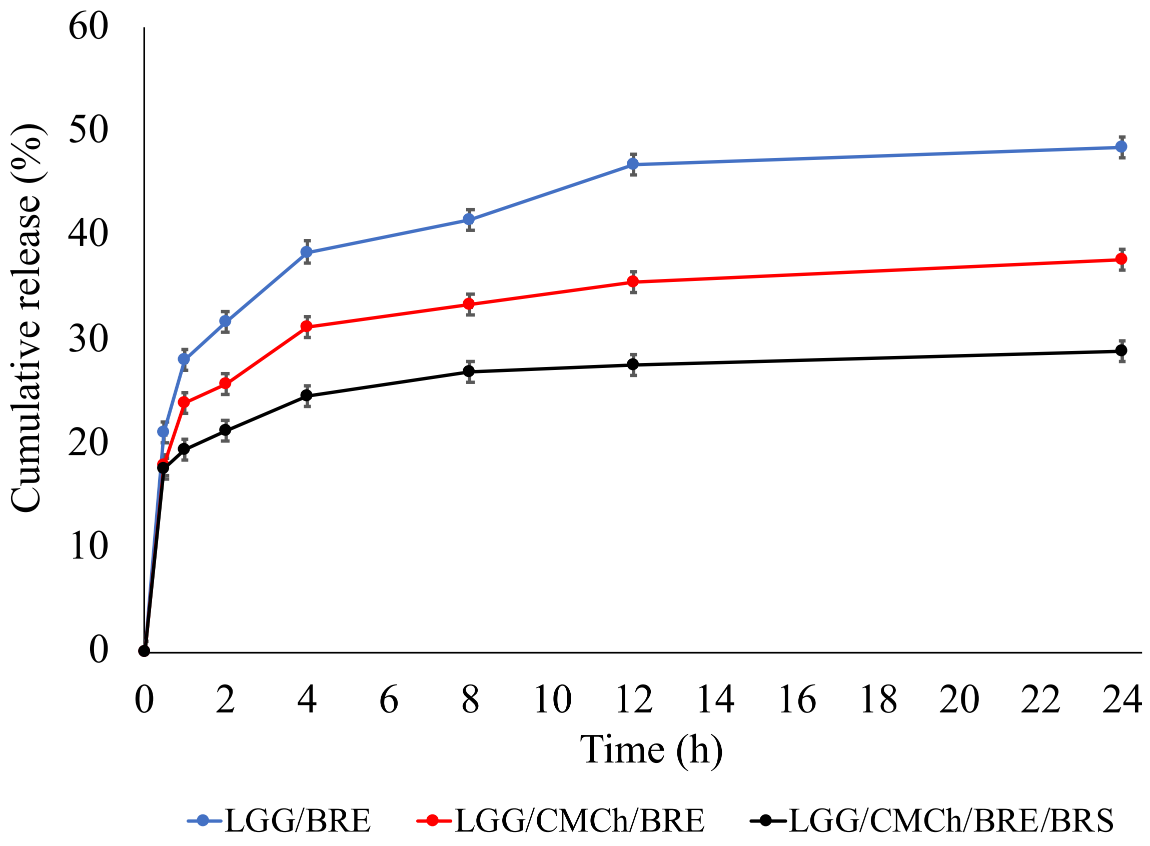

Figure S1. Time-dependent release rate of anthocyanins from LGG-based films in water at different times.
